# Response of Gut Microbiota to Dietary Fiber and Metabolic Interaction With SCFAs in Piglets

**DOI:** 10.3389/fmicb.2018.02344

**Published:** 2018-09-28

**Authors:** Boshuai Liu, Wenjing Wang, Xiaoyan Zhu, Xiao Sun, Junnan Xiao, Defeng Li, Yalei Cui, Chengzhang Wang, Yinghua Shi

**Affiliations:** ^1^College of Animal Science and Veterinary Medicine, Henan Agricultural University, Zhengzhou, China; ^2^Henan Key Laboratory of Innovation and Utilization of Grassland Resources, Zhengzhou, China

**Keywords:** dietary fiber, short-chain fatty acid, gut microbiota, diarrhea rate, piglets

## Abstract

Dietary fiber (DF) is increasingly thought to regulate diversity of piglet gut microbiota to alleviate weaning stress in piglets. This study was conducted to investigate the effects of DF on growth performance of piglets and composition of their gut microbiota, as well as the interaction between gut microbiota and short-chain fatty acids (SCFAs) in piglets. A total of 840 piglets were allocated to three dietary treatments consisting of a control group (CG), an alfalfa meal group (AG), and a commodity concentrated fiber group (OG) in a 30-day feeding trial. Gut mucosa and feces samples were used to determine bacterial community diversity by 16S rRNA gene amplicon sequencing. Fiber treatment had a positive effect on growth performance and metabolism of SCFAs in piglets, in particular, compared with CG, the diarrhea rate was significantly decreased, and the content of propionic acid (PA) in the cecum was markedly increased in AG. The Shannon indices of the jejunum microbiota in AG were higher than CG. At the genus level, compared to CG, in the duodenum, the relative abundance of *Paenibacillus* in AG and OG was higher; in the jejunum, the relative abundances of *Bacillus*, *Oceanobacillus*, *Paenibacillus*, *Lactococcus*, *Enterococcus*, and *Exiguobacterium* were higher, whereas the relative abundance of *Mycoplasma* was lower in AG; in the cecum, there was also lower relative abundance of *Helicobacter* in AG and OG, and furthermore, the relative abundance of *Faecalibacterium* in OG was higher than in CG and AG. Spearman correlation analysis showed that *Pseudobutyrivibrio* was positively correlated with acetic acid, PA, and butyric acid (BA), while *Bacteroides* and *Anaerotruncus* were negatively correlated with PA and BA. In addition, microbiota analyses among different intestine segments showed distinct differences in microbiota between the proximal and distal intestines. Bacteria in the proximal segments were mainly *Firmicutes*, while bacteria in the distal segments were mainly *Bacteroidetes* and *Firmicutes*. Overall, these findings suggested that DF treatment could reduce the diarrhea rate of piglets and had beneficial effects on gut health, which might be attributed to the alteration in gut microbiota induced by DF and the interaction of the gut microbiota with SCFAs.

## Introduction

The high mortality and low growth performance of piglets during the weaning period seriously affects production efficiency in the pig industry. Stress caused by changes in environment and feed as well as the immaturity of the digestive and immune systems results in a decrease in feed intake of piglets and also digestive disorders, leading to increased incidence of diarrhea in piglets (Lallès et al., 2007). Heo et al. (2013) reported that the occurrence of pathogenic diarrhea in piglets was mainly due to the release of endotoxin by *Escherichia coli* that adhered to gut epithelial cells. Breeders usually feed piglets with digestible diets, such as milk by-products, animal protein, and cooked rice, to prevent indigestible nutrients which are exploited by pathogens in hindgut (Mateos et al., 2006). Antibiotics have been recognized as one of the most successful therapies in medicine, and are administered in both human and veterinary medicine. However, the use of antibiotics is now debatable due to the growing number of antibiotic-resistant bacteria within human and animal gut microbiota. The application of antibiotics as part of the feed not only increases the cost of farming, but also fails to achieve the desired growth performance during the weaning period (Berrocoso et al., 2013). Therefore, there is an urgent need to find alternatives to antibiotics to both maintain piglet health at the critical weaning period and preserve public health.

At present, breeders are trying to relieve weaning stress of piglets through dietary fiber (DF). Studies have shown that DF influences the physicochemical properties of chyme (Canibe and Bach Knudsen, 2002), improving the morphology and micro-ecological environment of the gut, stimulating the secretion of digestive enzymes (Gerritsen et al., 2012), and promoting the development and integrity of the digestive tract mucosa (Liu et al., 2012). It has also been confirmed that DF contributes to the development, health, and the micro-ecological balance of piglet gut (Molist et al., 2010). Several studies have indicated that the effect of fiber on piglet diarrhea depends on the amount of fiber added and its physicochemical properties. Li and Zhang (2006) increased the content of crude fiber (CF) to 5.3% in the piglet diet by adding alfalfa meal and found that the fiber could reduce the diarrhea rate of piglets by affecting water absorption in the small intestine and inhibiting the growth of pathogens. Gut microbiota, serving as an important barrier in the host, play crucial roles in animal health and growth, including digestion and fermentation of carbohydrates, production of vitamins, maintenance of normal functions of the intestinal villi, regulation of the immune responses, and protection from pathogenic bacteria; however, gut microbiota also have effects on the liver, brain, and even on the metabolism of fat and muscle tissue, finally influencing the whole host metabolic network of nutrient and energy (Schroeder and Bäckhed, 2016). DF cannot be digested by host digestive enzymes but can be degraded into monosaccharides and short-chain fatty acids (SCFAs) by gut microbiota (Gresse et al., 2017). As the major energy source for hindgut microbiota, DF is believed to have significant effects on the composition and diversity of microbiota (Filippo and Hartl, 2010; Heinritz et al., 2016). Given that there are few studies evaluating the microbial mechanism of DF on weaning piglet health, the current research was undertaken to investigate the effects of different DF on growth performance, the composition and metabolites of gut and feces microbiota (GFM), as well as the metabolic interaction between hindgut microbiota and SCFAs in weaning piglets. This would provide some microbial mechanistic insights into the application of different DF on weaning piglet health.

## Materials and Methods

### Experimental Design and Sampling

All experimental procedures in this study were approved by the Institutional Animal Ethics Committee of Henan Agricultural University. A total of 840 piglets (Duroc × Landrace × Large White, age 35 days) with body weights of 9.26 ± 0.17 kg were randomly allotted to three treatments, with each treatment comprising four replicated pens of 70 piglets. The mixed groups of male and female piglets were housed in slatted floor indoor pens with access to feed and water *ad libitum* for 30 days under standard management conditions. All piglets were supplied with feed formulated to meet NRC (2012) recommendations. Experimental diets for each treatment were as follows: control group (CG) with corn and soybean-meal diet in which 2.27% CF was included; alfalfa meal group (AG) with diets in which CG diet was partly replaced with 5% alfalfa meal and the CF content was 3.27%; commodity concentrated fiber group (OG) with diets in which CG diet was partly replaced with 2% commodity concentrated fiber and the CF content was 3.27%. The detailed ingredient composition and nutrient content of the investigated diets are presented in **Table 1**. The average daily feed intake (ADFI), average daily gain (ADG), feed to gain (F:G), mortality, and diarrhea rate were recorded.

**Table 1:** Ingredient and nutrient composition (%, as feed) of the experimental diets.

Items	Diet^a^		
	CG	AG	OG
**Ingredient (%)**			
Corn	600.19	540.71	560.83
Soybean – puffed	120.00	120.00	120.00
Fermented soybean meal	50.00	50.00	50.00
Soybean meal	100.23	91.73	106.98
Alfalfa meal	–	50.00	–
Commodity concentrated fiber	–	–	20.00
Fish meal	40.00	40.00	40.00
Whey powder	50.00	50.00	50.00
Soybean oil	8.41	26.26	20.94
Salt	1.50	1.50	1.50
Limestone powder	6.77	6.77	6.77
Calcium hydrogen phosphate	5.66	5.66	5.66
Lysine, 98%	3.78	3.78	3.78
Methionine, 98%	1.20	1.33	1.28
Zinc oxide, 75%	2.20	2.20	2.20
Premix compound	10.00	10.00	10.00
Total	1000.00	1000.00	1000.00
**Nutrient composition**			
DE^b^, (MJ/kg)	14.64	14.64	14.64
CP^b^ (%)	19.00	19.00	19.00
CF^b^ (%)	2.27	3.38	3.37
Lys^b^ (%)	1.38	1.38	1.39
Met^b^ (%)	0.45	0.46	0.46
Ca^b^ (%)	0.75	0.82	0.75
P^b^ (%)	0.56	0.55	0.55


*^a^CG, control group; AG, alfalfa meal group; OG, commodity concentrated fiber group.*

*^*b*^DE, digestion energy; CP, crude protein; CF, crude fiber; Lys, lysine; Met, methionine; Ca, calcium; P, phosphorus.*

At the end of the experiment, one piglet in each replicate was selected for sampling/processing. Feces samples were obtained using sterilized equipment and were frozen in liquid nitrogen. After fasting overnight, the gastrointestinal tract of each piglet was removed immediately after slaughter and segments (duodenum, jejunum, ileum, cecum, and colon) were identified and ligated before separation. Sterile cold phosphate buffer was used to wash the mucosa. Subsequently, mucosal samples from the duodenum, jejunum, ileum, cecum, and colon were collected by scraping the mucosa with a sterile glass microscope slide. At the same time, samples of chyme from cecum and colon sections were also collected. All samples were stored in sterile cryopreservation tubes and immediately frozen at -80°C until further analysis. Then, three repeats were randomly selected from each treatment to be sequenced, one individual per replicate collecting six sample types (duodenum, jejunum, ileum, cecum, colon, and feces).

### DNA Extraction and 16S rRNA Gene Sequencing

Microbial DNA was extracted from the digestive tract (duodenum, jejunum, ileum, cecum, and colon) mucosa and feces samples using an E.Z.N.A^®^. Soil DNA Kit (Omega Bio-tek, Norcross, GA, United States) according to manufacturer’s protocols. The final DNA concentration and purity were determined by NanoDrop 2000 UV–vis spectrophotometer (Thermo Scientific, Wilmington, NC, United States), and DNA quality was checked by 1% agarose gel electrophoresis (Yang et al., 2015). The V3–V4 hypervariable regions of the bacterial 16S rRNA gene were amplified with primers 338F (5′-ACTCCTACGGGAGGCAGCAG-3′) and 806R (5′-GGACTACHVGGGTWTCTAAT-3′) by PCR (GeneAmp 9700, ABI, United States; Singh et al., 2015) using the following program: 3 min denaturation at 95°C; 27 cycles of 30 s at 95°C, 30 s annealing at 55°C, and 45 s elongation at 72°C; and a final extension at 72°C for 10 min. PCR reactions were performed in triplicate with each 20 μL reaction mixture containing 4 μL of 5× FastPfu Buffer, 2 μL of 2.5 mM dNTPs, 0.8 μL of each primer (5 μM), 0.4 μL FastPfu Polymerase, and 10 ng template DNA. The resulting PCR products were extracted from a 2% agarose gel and further purified using the AxyPrep DNA Gel Extraction Kit (Axygen Biosciences, Union City, CA, United States) and quantified using QuantiFluor^TM^-ST (Promega, United States) according to the manufacturer’s protocol.

### GenBank Accession Number

Purified amplicons were pooled in equimolar amounts and paired-end sequenced (2 × 300), on an Illumina MiSeq platform (Illumina, San Diego, CA, United States) according to standard protocols, by Majorbio Bio-Pharm Technology Co. Ltd. (Shanghai, China). The raw reads were deposited into the NCBI Sequence Read Archive (SRA) database under accession number SRP 121201.

### Bioinformatics Analysis of Sequencing Data

Raw fastq files were demultiplexed, quality-filtered by Trimmomatic, and merged by FLASH with the following criteria: (i) reads were truncated at any site receiving an average quality score < 20 over a 50-bp sliding window; (ii) primers were exactly matched allowing two nucleotide mismatching, and reads containing ambiguous bases were removed; and (iii) sequences whose overlap was longer than 10 bp were merged according to their overlap sequence. Operational taxonomic units (OTUs) were clustered with 97% similarity cutoff using UPARSE (version 7.1^1^), and chimeric sequences were identified and removed using UCHIME. The taxonomy of each 16S rRNA gene sequence was analyzed by RDP Classifier algorithm^2^ against the Silva (SSU123) 16S rRNA database using a confidence threshold of 70%. Biodiversity of the samples was calculated with ACE, Chao1, and Shannon indices (Schloss et al., 2009). The one-way analysis of molecular variance (AMOVA) method was used to identify differences between groups (Schloss et al., 2009). The shifts in the relative abundance of the bacterial phyla were displayed by a heatmap (Kolde, 2015), which was modeled with vegan package in R. Based on OTUs, Weighted-unifrac principal coordinate analysis (PCoA; Lozupone et al., 2011) and Bray–Curtis sample hierarchical cluster analysis were used to summarize the composition of gut microbiota in different parts of the digestive tract (Jiang et al., 2013). To determine the effect of posterior segment microbiota interacting with SCFAs, redundancy analysis (RDA) was performed at the genus level using the R language vegan packet (RDA 2014; Mu et al., 2017).

### Determination of SCFAs

The amount of SCFAs in cecum and colon contents were determined using gas chromatography (GC) according to the method of Alfa et al. (2017). The samples on a HP-88 column (100 m long × 0.25 mm diameter and 0.2 μm film thickness) were separated by using a TRACE^TM^ 1310 GC with flame ionization detector (FID). The temperature program was 70°C for 1 min, then raised to 180°C at 25°C/min and held for 1 min, then raised to 200°C at 10°C/min and held for 1 min, then raised to 220°C at 2°C/min and held for 10 min, and finally raised to 240°C at 20°C/min and held for 6 min. Samples were run with a 20:1 split ratio and a 1.3 mL/min column flow. Hydrogen was used as the carrier gas. The temperatures of the injector and detector were 270 and 290°C, respectively.

### Statistical Analysis

Statistical analyses were performed with the software SPSS 18.0 (IBM, New York, NY, United States). Data were evaluated by one-way ANOVA, and the differences between the means were assessed using Duncan’s test. *P* < 0.05 was considered statistically significant.

## Results

### Growth Performance and SCFA Fermentation

The effect of different DF on piglet growth performance is presented in **Table 2**. There were no differences in ADFI, ADG, and F:G among treatments (*P* > 0.05). Compared with CG treatment, the mortality rate of piglets in AG had a decreasing trend (*P* > 0.05), while the diarrhea rate decreased significantly (*P <* 0.05). The diarrhea rate of piglets in OG also had a decreasing trend (*P >* 0.05) compared with CG treatment.

**Table 2:** Effects of different DF on piglet^a^ growth performance.

Items	Diet		
	CG	AG	OG
ADFI^b^, g	589.46 ± 39.91	560.43 ± 47.74	556.37 ± 71.25
ADG^b^, g	352.66 ± 39.24	341.39 ± 50.17	327.07 ± 50.56
F:G^b^	1.68 ± 0.09	1.66 ± 0.15	1.71 ± 0.07
Mortality rate, %	4.3 ± 3.10	2.5 ± 2.94	3.9 ± 3.95
Diarrhea rate, %	1.4 ± 0.14^a^	0.9 ± 0.21^b^	1.2 ± 0.35^ab^


*^a^*N* = 4 for each treatment. Different superscript lowercase letters within each row mean significantly different (*P* < 0.05).*

*^b^ADFI, average daily feed intake; ADG, average daily gain; F:G, feed:gain.*

**FIGURE 1:**
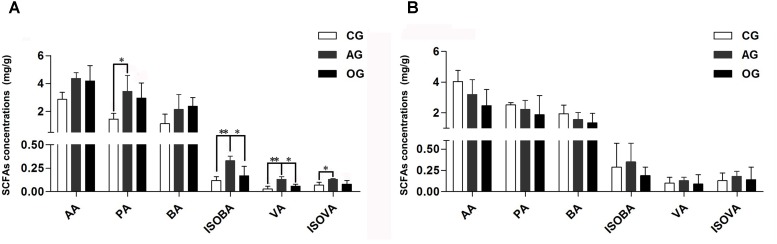
Effects of different DF on SCFAs fermentation of piglets. **(A)** SCFAs only cecum samples. **(B)** SCFAs only colon samples. AA, acetic acid; PA, propionic acid; BA, butyric acid; ISOBA, isobutyric acid; VA, valeric acid; ISOVA, isovaleric acid. ^∗^0.01 < *P* < = 0.05, ^∗∗^0.001 < *P* < = 0.01, ^∗∗∗^*P* < = 0.001.

Different DF greatly influences the amount of SCFAs in the cecum of piglets (**Figure 1A**). Compared with CG treatment, there was an increasing trend in the amounts of acetic acid (AA) and butyric acid (BA) in AG and OG, and the amounts of propionic acid (PA) and isovaleric acid (ISOVA) in AG significantly increased (*P <* 0.05). Furthermore, the amounts of valeric acid (VA) and isobutyric acid (ISOBA) in AG significantly increased compared to both CG and OG (*P <* 0.05). There were no significant differences observed in the amounts of SCFAs in the colon of piglets (*P >* 0.05; **Figure 1B**).

### Characterization of Microbiota Across the Gut and Feces

After removing incorrect and chimeric sequences, 2,033,322 sequencing reads were generated from the 54 samples. On average, 37,654 sequences per sample were obtained, with an average length of 443 bp. Using the criterion of 97% sequence similarity at the species level, 1751 OTUs were identified, all of which belonged to the bacteria domain according to Greengenes classification. Finally, on average, 417 ± 116 OTUs (Good’s coverage) per sample were identified. Rarefaction curves, Shannon curves, and Chao 1 curves were employed to analyze the richness and diversity of the microbiota community, as well as reflecting the data volume and sequencing depth (**Supplementary Figure S1**).

### Diversity and Composition of Gut Microbiota According to DF Treatment

**FIGURE 2:**
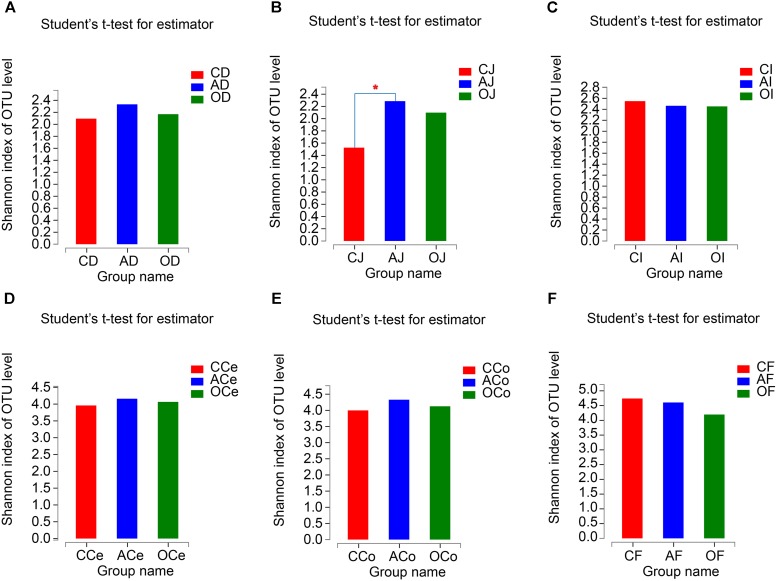
The Shannon Diversity Index Analyses of Microbiota Community. **(A)** Duodenum samples. **(B)** Jejunum samples. **(C)** Ileum samples. **(D)** Cecum samples. **(E)** Colon samples. **(F)** Feces samples. CD, AD, and OD: duodenum mucosal microbiota of control group, alfalfa meal group and commodity concentrated fiber group, respectively. CJ, AJ, and OJ: jejunum mucosal microbiota of control group, alfalfa meal group and commodity concentrated fiber group, respectively. CI, AI, and OI: ileum mucosal microbiota of control group, alfalfa meal group and commodity concentrated fiber group, respectively. CCe, ACe, and OCe: cecum mucosal microbiota of control group, alfalfa meal group and commodity concentrated fiber group, respectively. CCo, ACo, and OCo: colon mucosal microbiota of control group, alfalfa meal group and commodity concentrated fiber group, respectively. CF, AF, and OF: feces mucosal microbiota of control group, alfalfa meal group and commodity concentrated fiber group, respectively. ^∗^0.01 < *P* < = 0.05, ^∗∗^0.001 < *P* < = 0.01, ^∗∗∗^*P* < = 0.001.

From the analyses of the effects of different DF on the richness and diversity of GFM in piglets, there were no significant differences in Chao 1 indices of GFM (*P* > 0.05; **Supplementary Figure S2**). The Shannon indices of the jejunum microbiota (J) in AG increased significantly compared with CG treatment (*P* = 0.031), but no significant differences were observed in the other GFM (*P* > 0.05; **Figure 2**).

**FIGURE 3:**
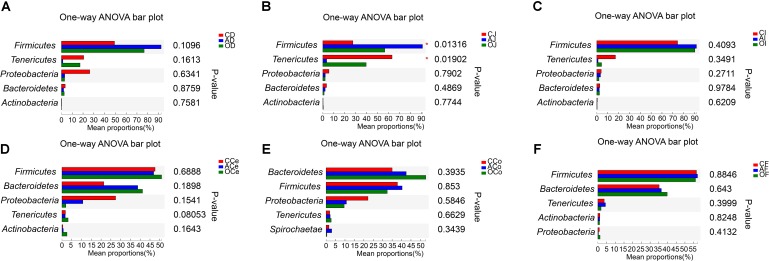
The Composition Analysis of Microbiota Community at the Phyla Level. **(A)** Duodenum samples. **(B)** Jejunum samples. **(C)** Ileum samples. **(D)** Cecum samples. **(E)** Colon samples. **(F)** Feces samples. CD, AD, and OD: duodenum mucosal microbiota of control group, alfalfa meal group and commodity concentrated fiber group, respectively. CJ, AJ, and OJ: jejunum mucosal microbiota of control group, alfalfa meal group and commodity concentrated fiber group, respectively. CI, AI, and OI: ileum mucosal microbiota of control group, alfalfa meal group and commodity concentrated fiber group, respectively. CCe, ACe, and OCe: cecum mucosal microbiota of control group, alfalfa meal group and commodity concentrated fiber group, respectively. CCo, ACo, and OCo: colon mucosal microbiota of control group, alfalfa meal group and commodity concentrated fiber group, respectively. CF, AF, and OF: feces mucosal microbiota of control group, alfalfa meal group and commodity concentrated fiber group, respectively. ^∗^0.01 < *P* < = 0.05, ^∗∗^0.001 < *P* < = 0.01, ^∗∗∗^*P* < = 0.001.

At the phylum level (**Supplementary Figure S3**), the most abundant bacteria were *Firmicutes*, *Bacteroidetes*, *Tenericutes*, *Proteobacteria*, *Actinobacteria*, and *Spirochaetes* across GFM, with the different phyla occupying different dominant positions across GFM. In the microbiota of the duodenum (D), jejunum (J), and ileum (I), *Firmicutes*, *Proteobacteria*, and *Tenericutes* were the dominant phyla. Among these bacteria, *Firmicutes* represented a ratio of 60–90%. *Bacteroidetes*, *Firmicutes*, and *Proteobacteria* were the dominant phyla in cecum (Ce) and colon (Co) microbiota where *Bacteroidetes* and *Firmicutes* occupied 30–50%, respectively. The effects of different DF on the microbiota community across GFM at the phylum level are presented in **Figure 3**. The relative abundance of *Firmicutes* in jejunum microbiota of the AG (AJ) increased significantly compared with that in the CG (CJ) and the commodity concentrated fiber group (OJ) (*P* = 0.002 and *P* = 0.035, respectively), while the relative abundance of *Tenericutes* in AJ decreased significantly compared with that in CJ (*P* = 0.007). No differences were observed in the microbiota community of D, I, Ce, Co, and F among treatments (*P* > 0.05).

**FIGURE 4:**
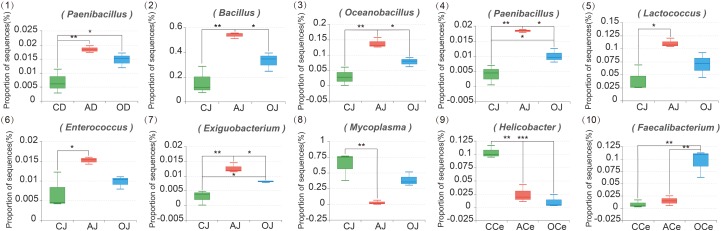
The Composition Analysis of Microbiota Community at the Genus Level. CD, AD, and OD: duodenum mucosal microbiota of control group, alfalfa meal group, and commodity concentrated fiber group, respectively. CJ, AJ, and OJ: jejunum mucosal microbiota of control group, alfalfa meal group, and commodity concentrated fiber group, respectively. CI, AI, and OI: ileum mucosal microbiota of control group, alfalfa meal group, and commodity concentrated fiber group, respectively. CCe, ACe, and OCe: cecum mucosal microbiota of control group, alfalfa meal group, and commodity concentrated fiber group, respectively. CCo, ACo, and OCo: colon mucosal microbiota of control group, alfalfa meal group, and commodity concentrated fiber group, respectively. CF, AF, and OF: feces mucosal microbiota of control group, alfalfa meal group, and commodity concentrated fiber group, respectively. ^∗^0.01 < *P* < = 0.05, ^∗∗^0.001 < *P* < = 0.01, ^∗∗∗^*P* < = 0.001.

At the genus level (**Supplementary Figure S4**), the relative abundance of the microbiota community across GFM also varied. A total of 15 bacterial genera were detected in D, J, and I where the relative abundance of these bacteria represented more than 1%; *Bacillus*, *Mycoplasma*, *Oceanobacillus*, and *Lactococcus* were the dominant genera. Moreover, 55 bacterial genera were detected in Ce, Co, and F where the relative abundance of these bacteria represented more than 1%. Among these bacteria, *Prevotella_9*, *Bacillus*, *Prevotellaceae_NK3B31_group*, and *Alloprevotella* were the dominant genera. The effects of different DF on the microbiota community at the genus level across GFM are presented in **Figure 4**. Different DF significantly affected the relative abundance of different bacterial genera across GFM. In the duodenum, the relative abundance of *Paenibacillus* in the AG (AD) and the commodity concentrated fiber group (OD) increased significantly compared with that in the CG (CD; *P* = 0.009 and *P* = 0.046, respectively). In the jejunum, different DF greatly influenced the relative abundance of *Bacillus*, *Oceanobacillus*, *Paenibacillus*, *Lactococcus*, *Enterococcus*, *Exiguobacterium*, and *Mycoplasma*; all these bacteria except for *Mycoplasma* presented higher relative abundance in AJ than that in CJ (*P* = 0.003, *P* = 0.003, *P* = 0.014, *P* < 0.001, *P* = 0.034, and *P* = 0.001, respectively). The relative abundances of *Bacillus*, *Oceanobacillus*, *Paenibacillus*, and *Exiguobacterium* in AJ were also much higher than those in OJ (*P* = 0.046, *P* = 0.040, *P* = 0.012, and *P* = 0.049, respectively). In addition, the relative abundances of *Paenibacillus* and *Exiguobacterium* in OJ were significantly higher than that in CJ (*P* = 0.048 and *P* = 0.028, respectively). However, the relative abundance of *Mycoplasma* in AJ decreased significantly compared with that in CJ (*P* = 0.007). In the cecum, the relative abundance of *Helicobacter* in the AG (ACe) and the commodity concentrated fiber group (OCe) decreased significantly compared with that in the CG (CCe) (both *P* < 0.001). Furthermore, the relative abundance of *Faecalibacterium* in OCe was much higher than that in CCe and ACe (*P* = 0.003 and *P* = 0.004, respectively).

**FIGURE 5:**
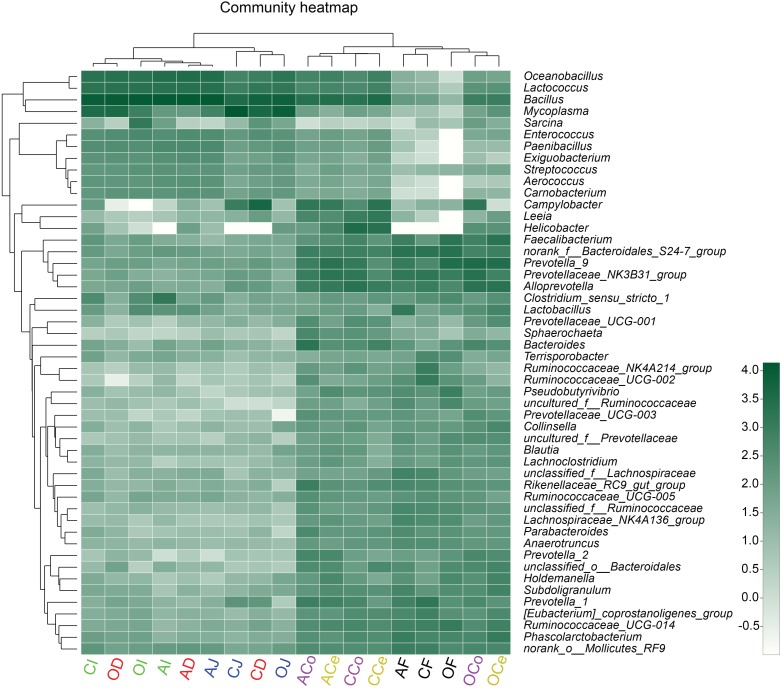
Heatmap of the microbiota composition in the gut and feces microbiota at the genus level. The relationship among species is determined by the complete clustering method with Bray–Curtis distance. The top tree showed the clustering relationship of samples. In the heatmap, green color means higher relative abundance whereas white color signifies lower relative abundance. CD, AD, and OD: duodenum mucosal microbiota of control group, alfalfa meal group, and commodity concentrated fiber group, respectively. CJ, AJ, and OJ: jejunum mucosal microbiota of control group, alfalfa meal group, and commodity concentrated fiber group, respectively. CI, AI, and OI: ileum mucosal microbiota of control group, alfalfa meal group, and commodity concentrated fiber group, respectively. CCe, ACe, and OCe: cecum mucosal microbiota of control group, alfalfa meal group, and commodity concentrated fiber group, respectively. CCo, ACo, and OCo: colon mucosal microbiota of control group, alfalfa meal group, and commodity concentrated fiber group, respectively. CF, AF, and OF: feces mucosal microbiota of control group, alfalfa meal group, and commodity concentrated fiber group, respectively.

To present the composition of the bacterial community directly, the relative abundance of the microbiota community was depicted by color intensity, and the community compositions were further clustered according to the similarity of species or relative abundance of samples, resulting in the heatmap presented in **Figure 5**. The microbiota in D, J, and I clustered together, as did the microbiota in Ce, Co, and F. It was observed that *Firmicutes* played a predominant role in D, J, and I while *Bacteroidetes* and *Firmicutes* were prevalent in Ce, Co, and F. In addition, the heatmap indicated that the diversity of microflora in Ce, Co, and F was much more varied than that in D, J, and I.

### Diversity and Composition of Gut Microbiota Across the GFM

From the richness and diversity analyses of microbiota community across the GFM of piglets, the Shannon and Chao 1 indices of proximal intestine (D, J, and I) microbiota decreased significantly compared with those of distal intestine and feces (Ce, Co, and F) microbiota (**Supplementary Figure S5**). Analysis of differences among the microbiota community across GFM at the phylum and genus level are presented in **Supplementary Figure S6**. There were differences among GFM; in particular, there were significant differences between small and large intestine microbiota. At the phylum level, compared to distal intestine, the relative abundance of *Firmicutes* in the proximal intestine was higher, while the relative abundance of *Bacteroidetes* was lower. At the genus level, compared to the distal intestine, the relative abundances of *Bacillus*, *Mycoplasma*, *Oceanobacillus*, and *Lactococcus* in the proximal intestine were higher, while the relative abundances of *Prevotella_9*, *Prevotellaceae_NK3B31_group*, *Alloprevotella*, *anorank_f_Bacteroidales_S24-7_group*, *Faecalibacterium*, *Prevotella_1*, *Ruminococcaceae_UCG-014*, *norank_o_Mollicutes_RF9*, and *Phascolarctobacterium* were lower. To further study similarities and differences among GFM, cluster tree and PCoA analysis was conducted (**Supplementary Figure S7**). It was clearly observed that the samples of the proximal intestine were distinct from those in the distal intestine.

### Association and Model Predictive Analysis

**FIGURE 6:**
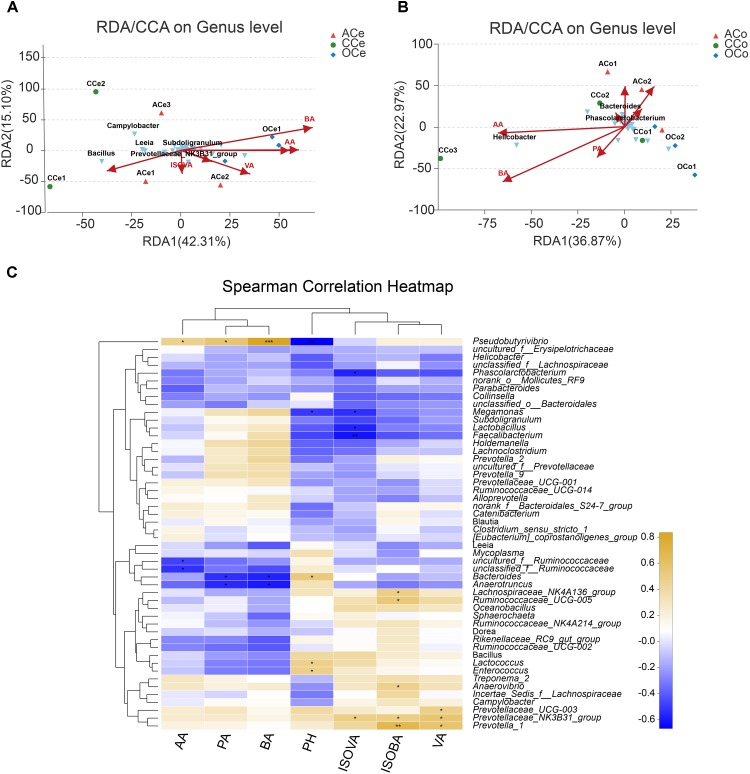
Association and model predictive analysis. **(A)** RDA targeting only cecum samples. **(B)** RDA targeting only colon samples. **(C)** Heatmap of the correlation analysis was conducted between the top 50 bacterial genera and the environmental factors. AA, acetic acid; PA, propionic acid; BA, butyric acid; ISOBA, isobutyric acid; VA, valeric acid; ISOVA, isovaleric acid. ^∗^0.01 < *P* < = 0.05, ^∗∗^0.001 < *P* < = 0.01, ^∗∗∗^*P* < = 0.001.

Redundancy analysis was conducted based on all samples of Ce and Co and their environmental factors (SCFAs, pH; **Figures 6A,B**). The correlation between microbiota distribution and environmental factors were as follows: BA > PA > AA > pH > VA > ISOVA > ISOBA in Ce, and BA > AA > PA > pH > ISOBA > VA > ISOVA in Co. In addition, RDA indicated that there was positive correlation among BA, PA, and AA, and negative correlation between pH and these three major SCFAs. Moreover, the correlation among these three major SCFAs is relatively tight in both Ce and Co. Correlation analysis was conducted between the top 50 bacterial genera and the environmental factors, and is directly reflected by a heatmap (**Figure 6C**). The threshold |*R*| > 0.4 is considered as having correlation. The results indicated that *Bacteroides* is positively correlated with pH, while *Pseudobutyrivibrio*, *Megamonas*, and *Lactobacillus* are negatively correlated with pH. *Pseudobutyrivibrio* is positively correlated with AA, while *uncultured_f_Ruminococcaceae* and *unclassified_f_Ruminococcaceae* are negatively correlated with this SCFA. *Pseudobutyrivibrio* is also positively correlated with PA and BA, but *Anaerotruncus* and *Bacteroides* are negatively correlated with them. *Prevotella_1*, *Ruminococcaceae_UCG-005*, *Prevotellaceae_NK3B31_group*, *Anaerovibrio*, and *Lachnospiraceae_NK4A136_group* are positively correlated with ISOBA.*Prevotella_1*, *Prevotellaceae_NK3B31_group*, and *Prevotellaceae_UCG-003* are positively correlated with VA. *Prevotellaceae_NK3B31_group* is positively correlated with ISOVA, but *Faecalibacterium*, *Phascolarctobacterium*, *Lactobacillus*, and *Megamonas* are negatively correlated with this SCFA.

## Discussion

Attempts have been made to add fiber to the diet of weaning piglets to make the piglets adapt quickly to the thicker feed during the growing season (Molist et al., 2014). However, some of the results concerning the effects of adding fiber to diets on growth performance in piglets were inconsistent (Freire et al., 2000; Hogberg and Lindberg, 2004; Jeaurond et al., 2008). Lindberg (2014) also confirmed that the type and origin of the fiber determined the effect of the fiber on the swine diet. In our study, the addition of fiber from different sources to piglet diets had no effects on ADFI, ADG, and F:G in piglets. However, compared to the CG, the diarrhea rate of piglets fed with alfalfa meal significantly reduced. From the perspective of fermentability of the fiber, alfalfa meal is rich in insoluble fiber (cellulose) but also has a little soluble fiber (pectins) (Brambillasca et al., 2015). Similarly, the commercial fiber used in this study is an insoluble, slowly fermented fiber. Gerritsen et al. (2012) reported that the addition of 15% insoluble non-starch polysaccharides (NSP) to the low-protein diet of piglets had no effect on the performance of piglets, but promoted development of the intestine and affected the colon microbiota. In addition, previous studies have shown that insoluble NSP can reduce the residence time of chyme in the gastrointestinal tract and alleviate the anorexia and digestive disorders of piglets caused by gut stasis, thereby reducing diarrhea in piglets (Molist et al., 2012). In the current study, the PA content in the cecum was markedly increased by alfalfa meal treatment, demonstrating that the fiber in the alfalfa meal appears to have a prebiotic effect. The main fermentation products of DF in the hindgut are SCFAs. Among them, AA, PA, and BA are the most abundant, comprising 90–95% of SCFAs present in the hindgut (Ríos-Covián et al., 2016). Fermentable fibers such as oligosaccharides and soluble NSP are usually fermented in the ileum, while the slowly fermented insoluble NSP can provide a growth medium for the microbiota until the end of the large intestine. It is well known that SCFAs play an important role in maintaining the morphology and function of epithelial cells. Butyrate is metabolized by hindgut cells as the main energy source, and the rest can be transported to the liver and used in different biosynthetic pathways. Propionate is mainly involved in the process of gluconeogenesis (Zhang Q. et al., 2016), while acetate and butyrate are predominantly involved in lipid biosynthesis (den Besten et al., 2013; Ríos-Covián et al., 2016). In addition, SCFAs act as specific G protein-coupled receptor (GPR) signaling molecules and are involved in the regulation of glucose and lipid metabolism (den Besten et al., 2013). Furthermore, Shibata et al. (2017) reported that SCFAs can decrease pH of the gut and inhibit the colonization and growth of some pathogens such as *E. coli* and *Salmonella*. The findings in the present study indicated that alfalfa meal treatment could generate more SCFAs and promote gut health of the piglets, which are closely correlated with the decreased diarrhea rate of piglets in the alfalfa meal treatment.

With the rapid development of sequencing technology in recent years, more researchers are using Illumina MiSeq sequencing which is lower in cost and has a higher sequencing depth and coverage than 454 pyrosequencing (Fouhy et al., 2015). In this study, we employed Illumina MiSeq sequencing to investigate the effects of different DF on the richness and diversity across GFM, and found that different DF had varied influences on microbiota of the same intestinal segment. In general, Chao1 and Shannon indices of GFM showed an increasing trend under the fiber treatment. In particular, the Shannon index of the jejunum microbiota was significantly increased by alfalfa meal treatment, indicating that fiber treatment could increase the richness and diversity of GFM, and subsequently promote the health of piglets. Gresse et al. (2017) also reported that the addition of a certain amount of fiber to the basal diet of piglets could increase the diversity of GFM.

Dietary fiber plays an important role in maintaining gut microbiota balance and gut health. As Castillo et al. (2007) reported, the host gut microbiota vary in response to the composition of DF due to the specific substrate preference of the bacteria. Therefore, the composition of gut microbiota could be regulated by supplementation with specific DF. Zhang D. et al. (2016) have shown that fiber from alfalfa sources in piglet diets can regulate gut microbiota and promote gut health. In this study, analysis of microbiota data at phylum level showed that alfalfa meal treatment significantly increased the relative abundance of *Firmicutes* and decreased the relative abundance of *Tenericutes* in the jejunum. These changes seemed to have a positive effect on gut health of piglets. This may be explained by the fact that *Firmicutes* mainly includes beneficial bacteria, while *Tenericutes* mainly includes harmful bacteria. Further analysis at the genus level indicated that DF treatment significantly increased the relative abundance of *Paenibacillus*, *Bacillus*, *Oceanobacillus*, *Lactococcus*, *Enterococcus*, *Exiguobacterium*, and *Faecalibacterium*, while significantly decreasing the relative abundance of *Helicobacter* and *Mycoplasma*. Numerous studies have reported on the role of these bacterial genera in gut health. Members of the genus *Paenibacillus* are able to produce antibacterial and antifungal compounds against plant and animal pathogens (Youngryun et al., 2000). *Bacillus*, *Lactococcus*, and *Enterococcus* *faecalis* have beneficial effects on reducing mortality, forming antimicrobial compounds, inhibiting the growth of pathogens, and regulating activation of the immune system (Lee and Kim, 2011; Hu et al., 2015). *Faecalibacterium* is involved in butyrate synthesis, which is the main nutrient for the regeneration and repair of gut epithelial cells (Lopezsiles et al., 2017). However, infection with *Helicobacter* is one of the major inducers of chronic active gastritis and peptic ulcer (Heimesaat et al., 2014). The family *Mycoplasmataceae* is known to infect pigs (Deblanc et al., 2016). In this study, there was a high relative abundance of *Mycoplasma sualvi* and *Helicobacter* in the pigs consuming control feed, which might result in gut inflammation, and subsequently piglet diarrhea. In conclusion, alteration in gut microbiota induced by DF treatment resulted in an improvement in gut health, potentially explaining the reduction of piglet diarrhea.

Interestingly, DF could regulate the gut microbiota on one hand, while on the other hand, some bacteria were also involved in the metabolic processing of DF. In the current study, the amounts of *Exiguobacterium* and *Paenibacillus* in fiber treatment significantly increased. *Exiguobacterium* plays key roles in the fermentation of cellulose and the transglycosylation of sugars because of its high β-glucosidase activity (Gao et al., 2015). *Paenibacillus* sp. HY-8 has high activity of GH11β-1,4-xylanase and extracellular endo-β-1,4-mannanase, promoting the degradation of xylan and mannan (Heo et al., 2006; Kim et al., 2017). Similarly, Zhao et al. (2018) found that high insoluble DF increased the relative abundance of fiber-degrading bacteria in pig feces. Further study on the correlation analysis of fermentation products of DF (SCFAs) and microbiota could provide additional insights into their interactions.

From the perspective of the diversity of microbiota in different parts of the intestines, this study indicated that the gut microbiota varied greatly between proximal and distal intestines, but the difference between adjacent intestinal segments was relatively small. In the proximal intestine, the predominant microbiota was *Firmicutes*, mainly containing *Bacillus*, *Mycoplasma*, *Oceanobacillus*, *Lactococcus*, and other organisms. For the distal intestine, the prevailing microbiota were *Bacteroidetes* and *Firmicutes*, including*Prevotella_9*, *Prevotellaceae_NK3B31_group*, *Alloprevotella*,*anorank_f_Bacteroidales_S24-7_group*, *Faecalibacterium*, *Prevotella_1*, *Ruminococcaceae_UCG-014*, *norank_o_Mollicutes_RF9*, *Phascolarctobacterium*, and so on. These results were consistent with the distribution of gut microbiota of other species (Mu et al., 2017). The observed differences in microbiota between proximal and distal intestines were closely related to the function and environment of different intestine segments. The function of the proximal segment is mainly digestion and absorption of protein, fat, and starch, while the hindgut is a strictly anaerobic environment; therefore, the majority of bacteria colonizing the posterior segment are strict anaerobes and facultative anaerobes. DF can be decomposed by microbiota fermentation in the hindgut to generate SCFAs as energy for epithelial cells, which was in agreement with the increase of SCFAs in the cecum and colon.

The micro-ecosystem of the posterior segment of the pig gut is very complicated as approximately 400–500 species and about 10^12^ microbiota per gram of intestine exist in this segment (Castillo et al., 2007). To date, studies have mainly focused on the species and characteristics of porcine posterior hyphae-degrading bacteria (Shuchi et al., 2013; Christopherson et al., 2014), and there is limited research on the correlation between hindgut fiber-degrading bacteria and SCFAs generated by fiber fermentation. Taking into account that specific microbiota in the gut may be involved in the digestion and metabolism of the fiber, further investigations were performed in the current study to analyze the metabolic interaction of gut microbiota with fermentation products of DF in the hindgut. Using RDA/CCA analysis, AA, PA, and BA were significantly associated with cecum and colon microbiota. Furthermore, Spearman correlation analysis on the top 50 microbiota genera and the environmental factors in the hindgut showed that the microbiota bacteria with positive effects on SCFAs were *Pseudobutyrivibrio*, *Ruminococcaceae_UCG-005*, *Prevotellaceae_NK3B31_group*, *Lachnospiraceae_NK4A136_group*, *Anaerovibrio*, *Prevotellaceae_ UCG-003*, and so on, while the microbiota negatively correlated with SCFAs were *Bacteroides* and *Anaerotruncus*. Previous research found that *Bacteroides* are the most prevalent anaerobic bacteria in the hindgut. *Bacteroides fragilis* comprises only 2% of the total *Bacteroides* in the gut but causes more than 70% of *Bacteroides* infections. In contrast, *Pseudobutyrivibrio* decomposes various carbohydrates, mainly generating BA as well as formic acid and lactic acid (Zhang et al., 2017). The metabolic process of the family *Ruminococcaceae* is a typical type of mixed fermentation, producing AA, formic acid, and a small amount of lactic acid (Biddle et al., 2013). The main fermentation products generated by *Prevotella* are AA, succinic acid, a small amount of ISOBA, ISOVA, and lactic acid (Kovatcheva-Datchary et al., 2015). All of these investigations in our study suggested that specific bacteria in the hindgut were involved in the process of digestion and metabolism of the DF, subsequently affecting generation of SCFAs and inhibiting colonization of some harmful bacteria, and finally possibly influencing the whole network of nutrient and energy metabolism, which was in agreement with the reports of Lin et al. (2017). However, the interaction of gut microbiota with SCFAs and their response mechanism to DF requires further study.

## Conclusion

Diets containing alfalfa meal and commodity concentrated fiber had positive effects on growth performance and SCFAs metabolism of piglets. In particular, the diarrhea rate of piglets was significantly decreased, and the content of PA in the cecum was markedly increased by AG treatment. Furthermore, DF had beneficial effects on bacterial richness and diversity, as well as gut microbiota composition. Piglets with fiber treatment had greater proportions of beneficial bacteria and fiber-degrading bacteria, whereas harmful bacteria were significantly decreased. Further investigation indicated that there were close metabolic interactions between hindgut microbiota and SCFAs. These results suggested that DF reduced the diarrhea rate of piglets and had prebiotic effects on gut health, which might be attributed to the alteration in gut microbiota induced by DF and their interaction with SCFAs. In addition, the microbiota composition of gut and feces samples in piglets was systematically analyzed to provide theoretical support for the findings of microbiota differences between the proximal and distal intestines. These findings will facilitate the development of strategies for the regulation of gut microbiota through DF to improve piglet health and productivity.

## Ethics Statement

This study was performed strictly according to the recommendations of the Guide for the Care and Use of Laboratory Animals of the Ministry of Health, China. The research protocol was reviewed and approved by the Research Ethics Committee of Henan Agricultural University. Permission was obtained from the farm owners before the samples were collected. Not involved in these situations.

## Author Contributions

YS conceived and designed the experiments. BL, WW, and XZ performed the experiments and wrote the paper. XS and JX analyzed the data. DL, YC, and CW contributed reagents, materials, and analysis tools.

## Conflict of Interest Statement

The authors declare that the research was conducted in the absence of any commercial or financial relationships that could be construed as a potential conflict of interest.

## Footnotes

1 http://drive5.com/uparse/

2 http://rdp.cme.msu.edu/
