# Supplementary material for: Response of Gut Microbiota to Dietary Fiber and Metabolic Interaction With SCFAs in Piglets
Source: Front Microbiol. 2018 Sep 28;9:2344. doi: 10.3389/fmicb.2018.02344 (PMC6172335; doi:10.3389/fmicb.2018.02344)
Supplement: Supplementary file 1 [file Data_Sheet_1.ZIP › 20180827_SHI_Supplementary_Material/20180827_SHI_Supplementary_Material.docx]

Supplementary Material

Response of Gut Microbiota to Dietary Fiber and Metabolic Interaction with SCFAs in Piglets

Boshuai Liu^1^, Wenjing Wang^1^, Xiaoyan Zhu^1,2^, Xiao Sun^1^, Junnan Xiao^1^, Defeng Li^1,2^, Yalei Cui^1,2^, Chengzhang Wang^1,2^ and Yinghua Shi^1,2,^*

*Correspondence:

Yinghua Shi

[annysyh@henau.edu.cn](mailto:annysyh@henau.edu.cn)

# Supplementary Figures


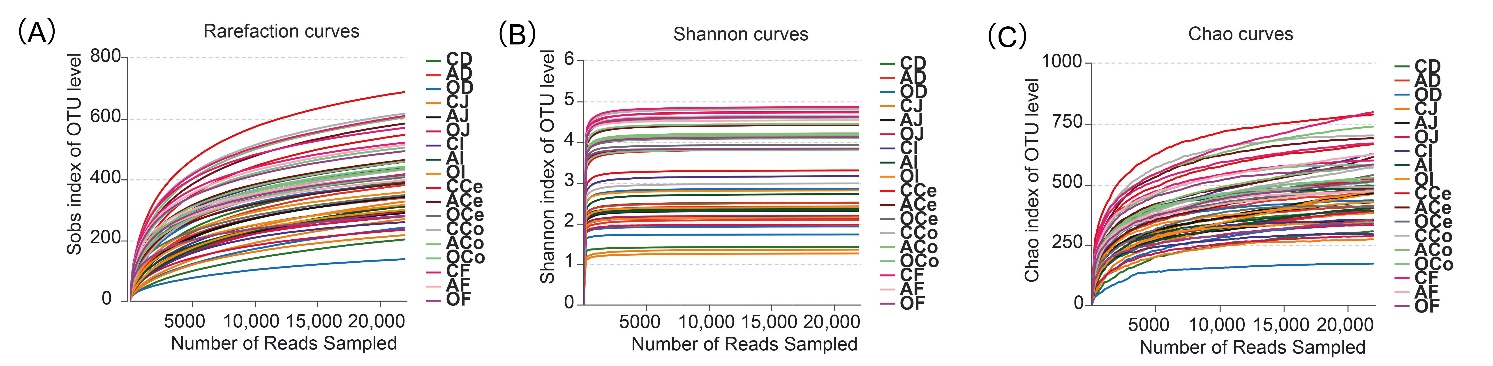


**Figure S1. Alpha diversities (A~C). (The operational taxonomic units (OTUs) were defined with 3% dissimilarity level).** (A) Abundance of actual observations (Sobs), (B) The diversity index (Shannon), (C) The richness estimators (Chao 1). Abbreviations: CD, AD, OD: duodenum mucosal microbiota of control group, alfalfa meal group and commodity concentrated fiber group, respectively. CJ, AJ, OJ: jejunum mucosal microbiota of control group, alfalfa meal group and commodity concentrated fiber group, respectively. CI, AI, OI: ileum mucosal microbiota of control group, alfalfa meal group and commodity concentrated fiber group, respectively. CCe, ACe, OCe: cecum mucosal microbiota of control group, alfalfa meal group and commodity concentrated fiber group, respectively. CCo, ACo, OCo: colon mucosal microbiota of control group, alfalfa meal group and commodity concentrated fiber group, respectively. CF, AF, OF: feces mucosal microbiota of control group, alfalfa meal group and commodity concentrated fiber group, respectively.


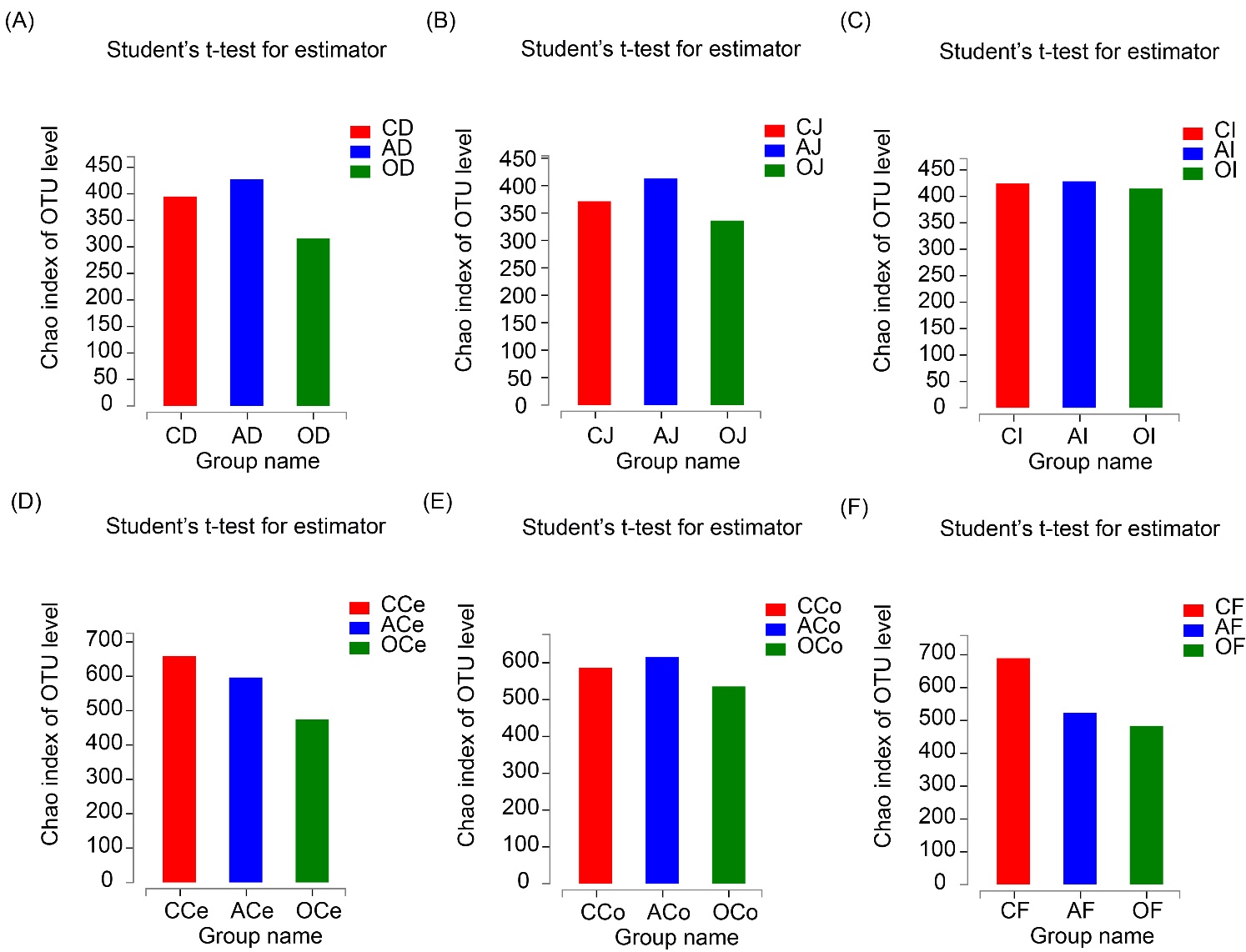
**Figure S2. The Chao 1 Richness Estimators Analyses of Microbiota Community (A~E) (The operational taxonomic units (OTUs) were defined with 3% dissimilarity level).** Abbreviations: CD, AD, OD: duodenum mucosal microbiota of control group, alfalfa meal group and commodity concentrated fiber group, respectively. CJ, AJ, OJ: jejunum mucosal microbiota of control group, alfalfa meal group and commodity concentrated fiber group, respectively. CI, AI, OI: ileum mucosal microbiota of control group, alfalfa meal group and commodity concentrated fiber group, respectively. CCe, ACe, OCe: cecum mucosal microbiota of control group, alfalfa meal group and commodity concentrated fiber group, respectively. CCo, ACo, OCo: colon mucosal microbiota of control group, alfalfa meal group and commodity concentrated fiber group, respectively. CF, AF, OF: feces mucosal microbiota of control group, alfalfa meal group and commodity concentrated fiber group, respectively. * 0.01 < *P* <= 0.05, ** 0.001 < *P* <= 0.01, *** *P* <= 0.001.


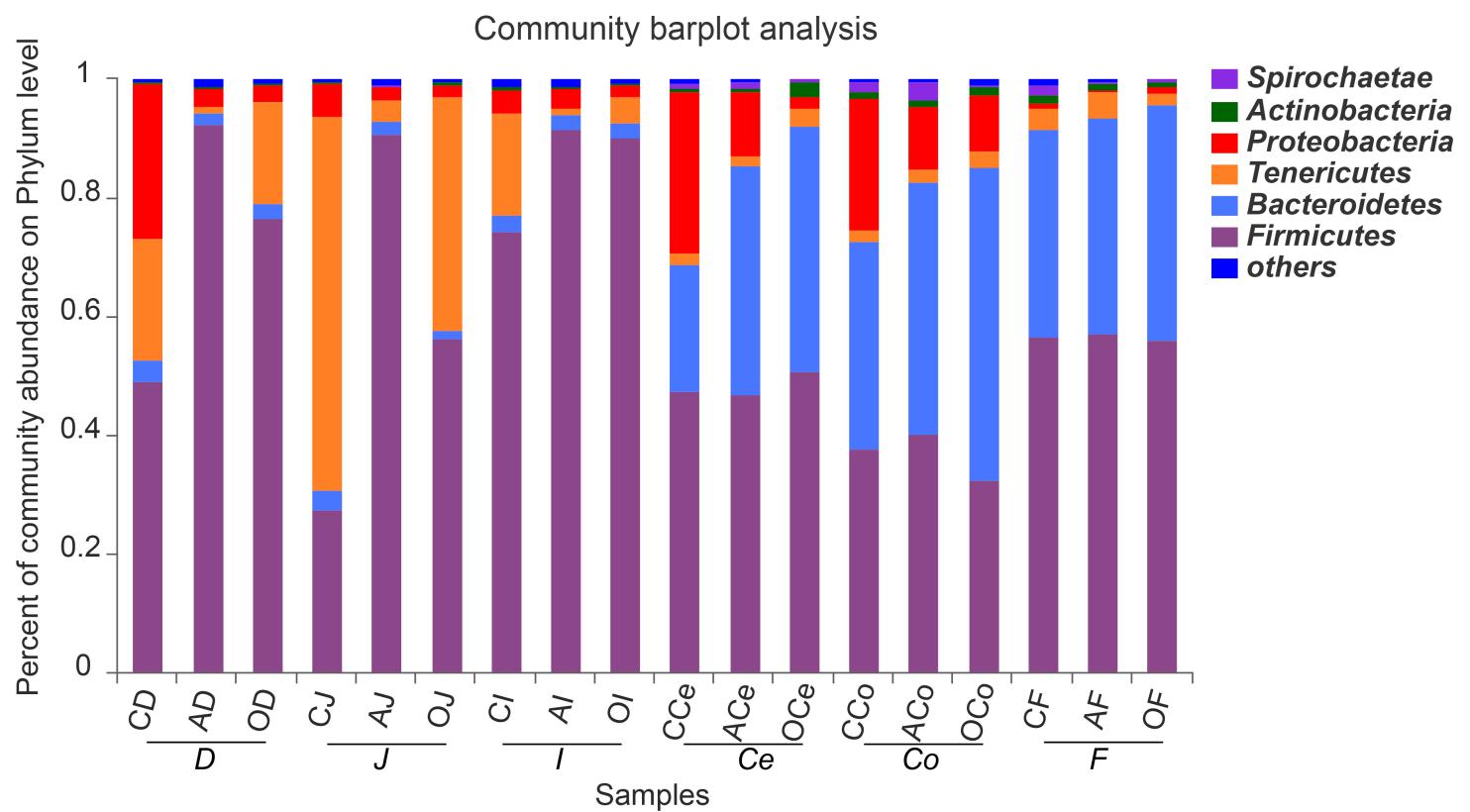
 **Figure S3. Relative abundances of the dominant bacterial phyla (**Microbiota community bar plot at the phyla level with the relative abundance was higher than 1%). Abbreviations: D: duodenum, J: jejunum, I: ileum, Ce: cecum, Co: colon, F: feces; CD, AD, OD: duodenum mucosal microbiota of control group, alfalfa meal group and commodity concentrated fiber group, respectively. CJ, AJ, OJ: jejunum mucosal microbiota of control group, alfalfa meal group and commodity concentrated fiber group, respectively. CI, AI, OI: ileum mucosal microbiota of control group, alfalfa meal group and commodity concentrated fiber group, respectively. CCe, ACe, OCe: cecum mucosal microbiota of control group, alfalfa meal group and commodity concentrated fiber group, respectively. CCo, ACo, OCo: colon mucosal microbiota of control group, alfalfa meal group and commodity concentrated fiber group, respectively. CF, AF, OF: feces mucosal microbiota of control group, alfalfa meal group and commodity concentrated fiber group, respectively.


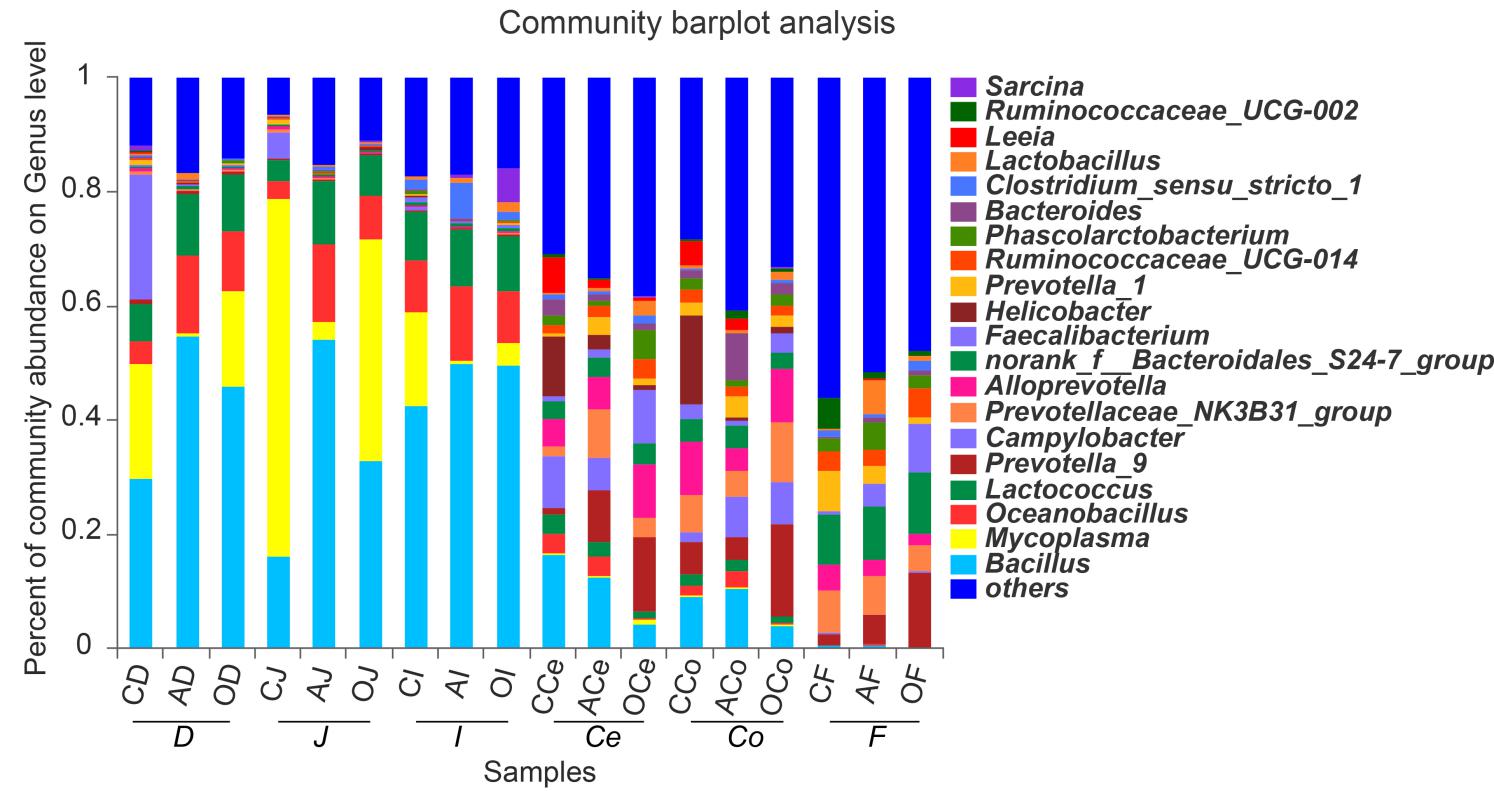
 **Figure S4. Relative abundances of the dominant bacterial genus (**Microbiota community bar plot at the genus level with the relative abundance was higher than 5%**).** Abbreviations: D: duodenum, J: jejunum, I: ileum, Ce: cecum, Co: colon, F: feces; CD, AD, OD: duodenum mucosal microbiota of control group, alfalfa meal group and commodity concentrated fiber group, respectively. CJ, AJ, OJ: jejunum mucosal microbiota of control group, alfalfa meal group and commodity concentrated fiber group, respectively. CI, AI, OI: ileum mucosal microbiota of control group, alfalfa meal group and commodity concentrated fiber group, respectively. CCe, ACe, OCe: cecum mucosal microbiota of control group, alfalfa meal group and commodity concentrated fiber group, respectively. CCo, ACo, OCo: colon mucosal microbiota of control group, alfalfa meal group and commodity concentrated fiber group, respectively. CF, AF, OF: feces mucosal microbiota of control group, alfalfa meal group and commodity concentrated fiber group, respectively.


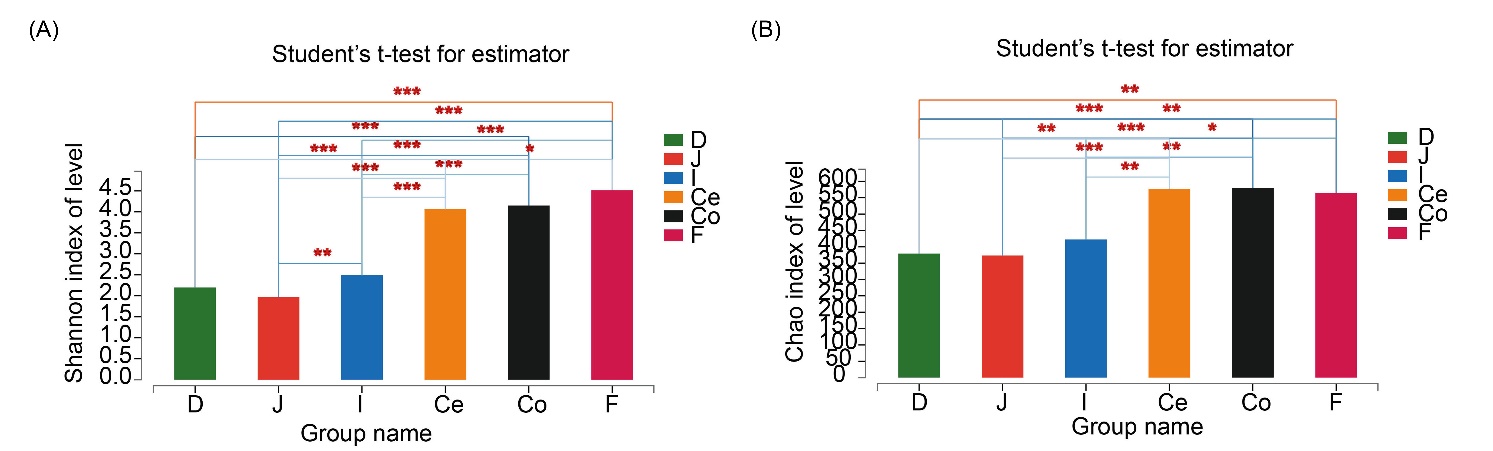
 **Figure S5. Alpha Diversity Statistics Comparison.** (A) The Shannon diversity index analyses of microbiota community across the gut and feces microbiota (GFM) of piglet; (B) The Chao1 estimator analyses of microbiota community across the GFM of piglet. Abbreviations: D: duodenum, J: jejunum, I: ileum, Ce: cecum, Co: colon, F: feces; CD, AD, OD: duodenum mucosal microbiota of control group, alfalfa meal group and commodity concentrated fiber group, respectively. CJ, AJ, OJ: jejunum mucosal microbiota of control group, alfalfa meal group and commodity concentrated fiber group, respectively. CI, AI, OI: ileum mucosal microbiota of control group, alfalfa meal group and commodity concentrated fiber group, respectively. CCe, ACe, OCe: cecum mucosal microbiota of control group, alfalfa meal group and commodity concentrated fiber group, respectively. CCo, ACo, OCo: colon mucosal microbiota of control group, alfalfa meal group and commodity concentrated fiber group, respectively. CF, AF, OF: feces mucosal microbiota of control group, alfalfa meal group and commodity concentrated fiber group, respectively. * 0.01 < *P* <= 0.05, ** 0.001 < *P* <= 0.01, *** *P* <= 0.001.


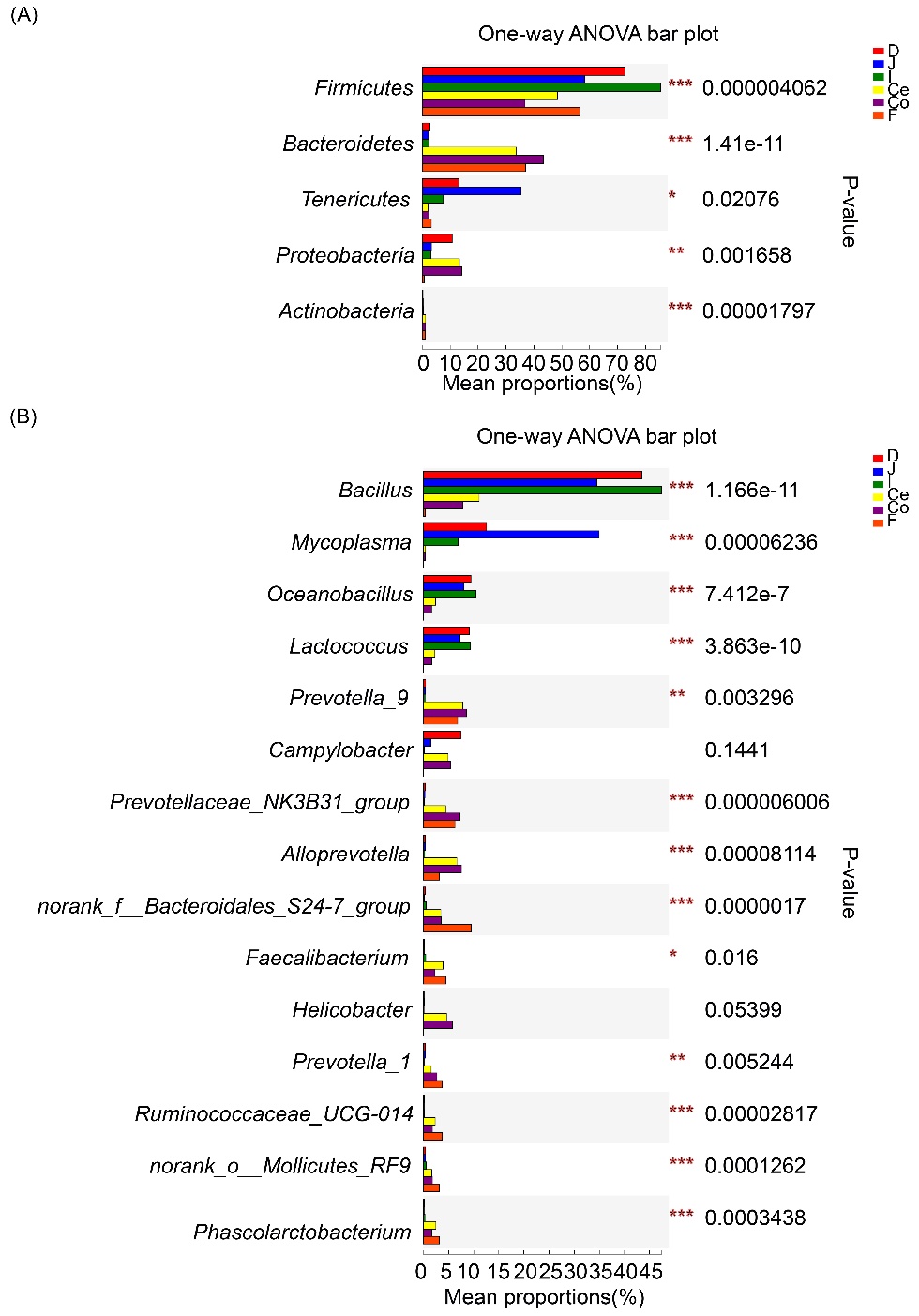
**Figure S6. Analysis of differences among the microbiota community across gut and feces microbiota** (GFM)**.** (A) Analysis of difference among microbiota community across GFM at the phyla level. (B) Analysis of difference among the top 15 microbiota community across GFM at the genus level. Abbreviations: D: duodenum, J: jejunum, I: ileum, Ce: cecum, Co: colon. * 0.01 < *P* <= 0.05, ** 0.001 < *P* <= 0.01, *** *P* <= 0.001.


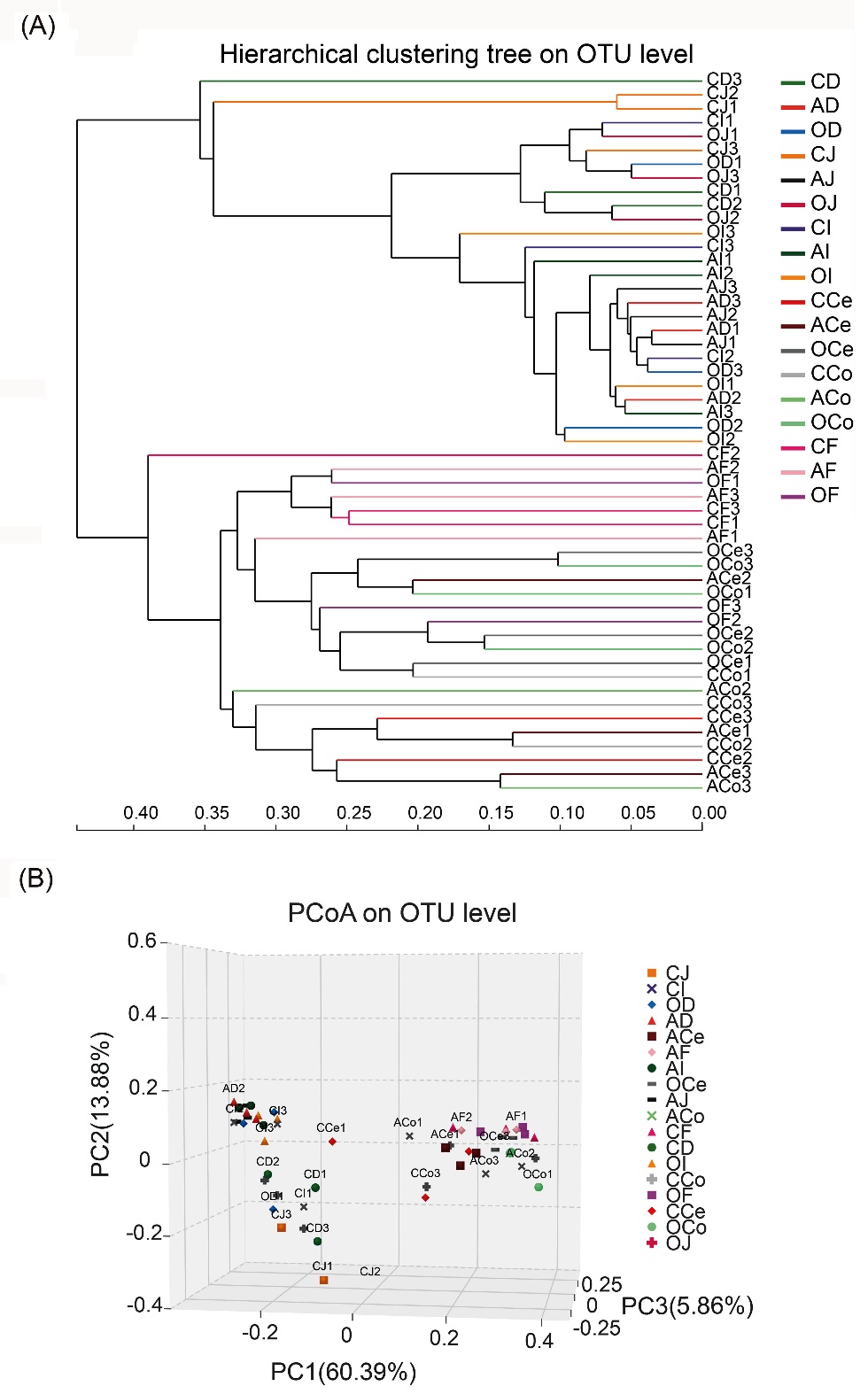
**Figure S7. Beta diversity analysis.** (A) Bray-Curtis dissimilarity based dendrogram showing the clustering of samples at the OTU level; (B) Weighted UniFrac PCoA of the microbiota. Each symbol and color represents each gut location microbiota.
