# Supplementary figures and images for: Response of Gut Microbiota to Dietary Fiber and Metabolic Interaction With SCFAs in Piglets
Source: Front Microbiol. 2018 Sep 28;9:2344. doi: 10.3389/fmicb.2018.02344 (PMC6172335; doi:10.3389/fmicb.2018.02344)

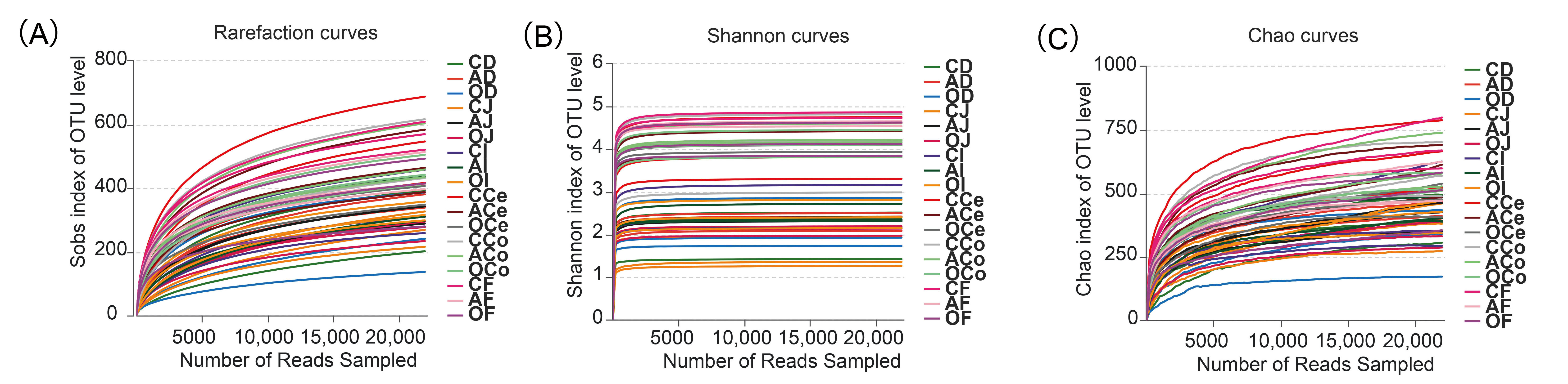

Supplement: Supplementary file 1 [file Data_Sheet_1.ZIP › 20180827_SHI_Supplementary_Material/supplementary_Figure/Figure S1.jpg]

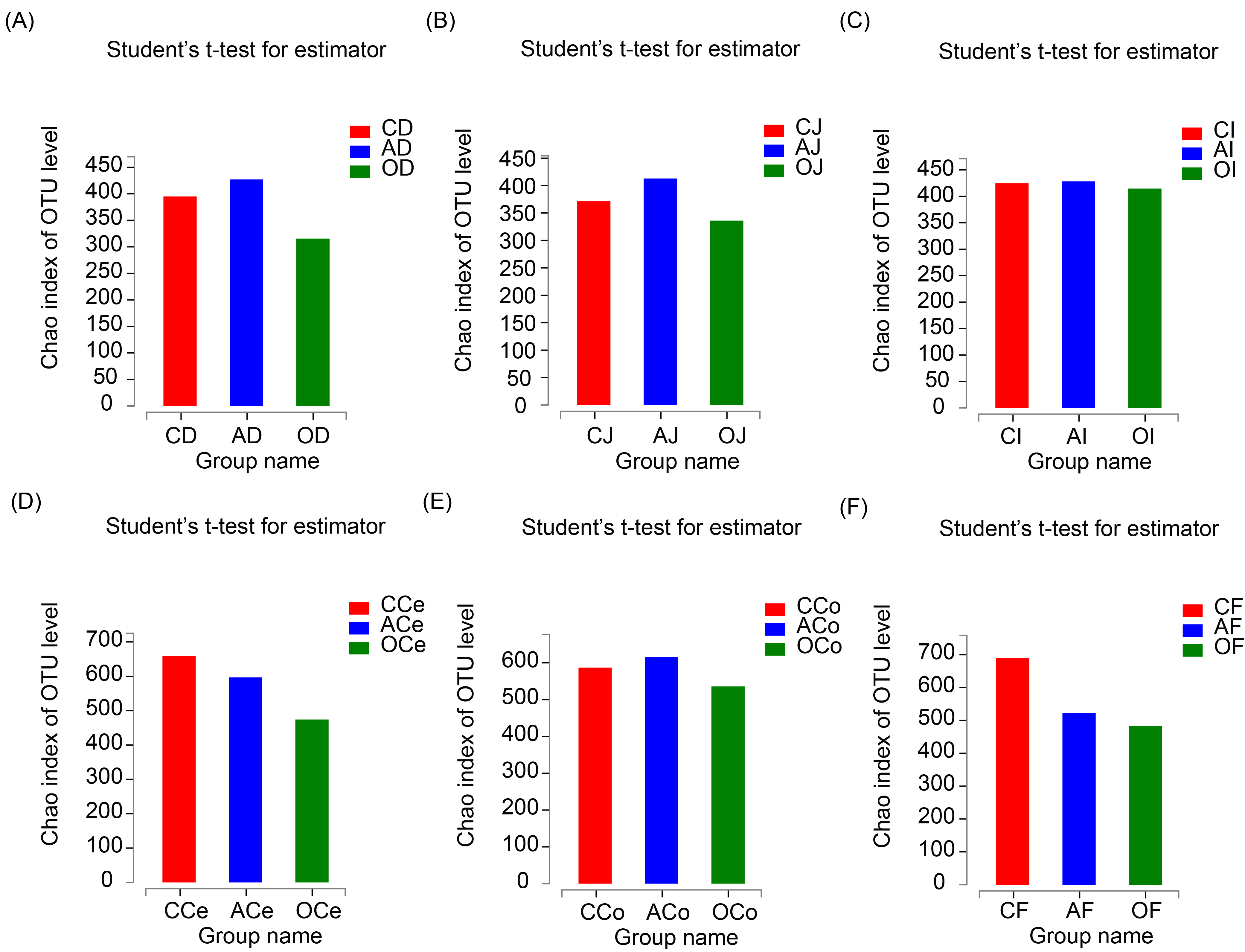

Supplement: Supplementary file 1 [file Data_Sheet_1.ZIP › 20180827_SHI_Supplementary_Material/supplementary_Figure/Figure S2.jpg]

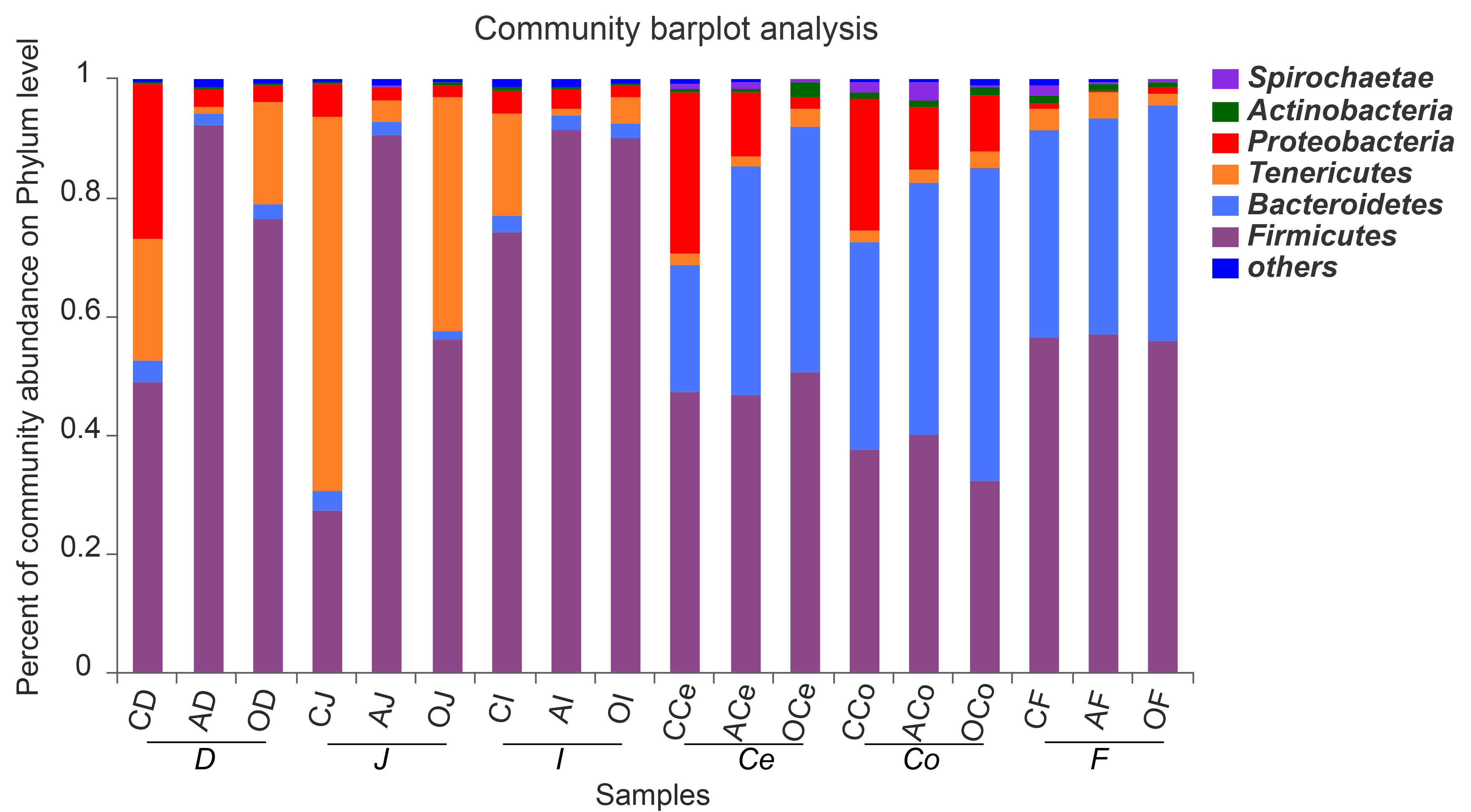

Supplement: Supplementary file 1 [file Data_Sheet_1.ZIP › 20180827_SHI_Supplementary_Material/supplementary_Figure/Figure S3.jpg]

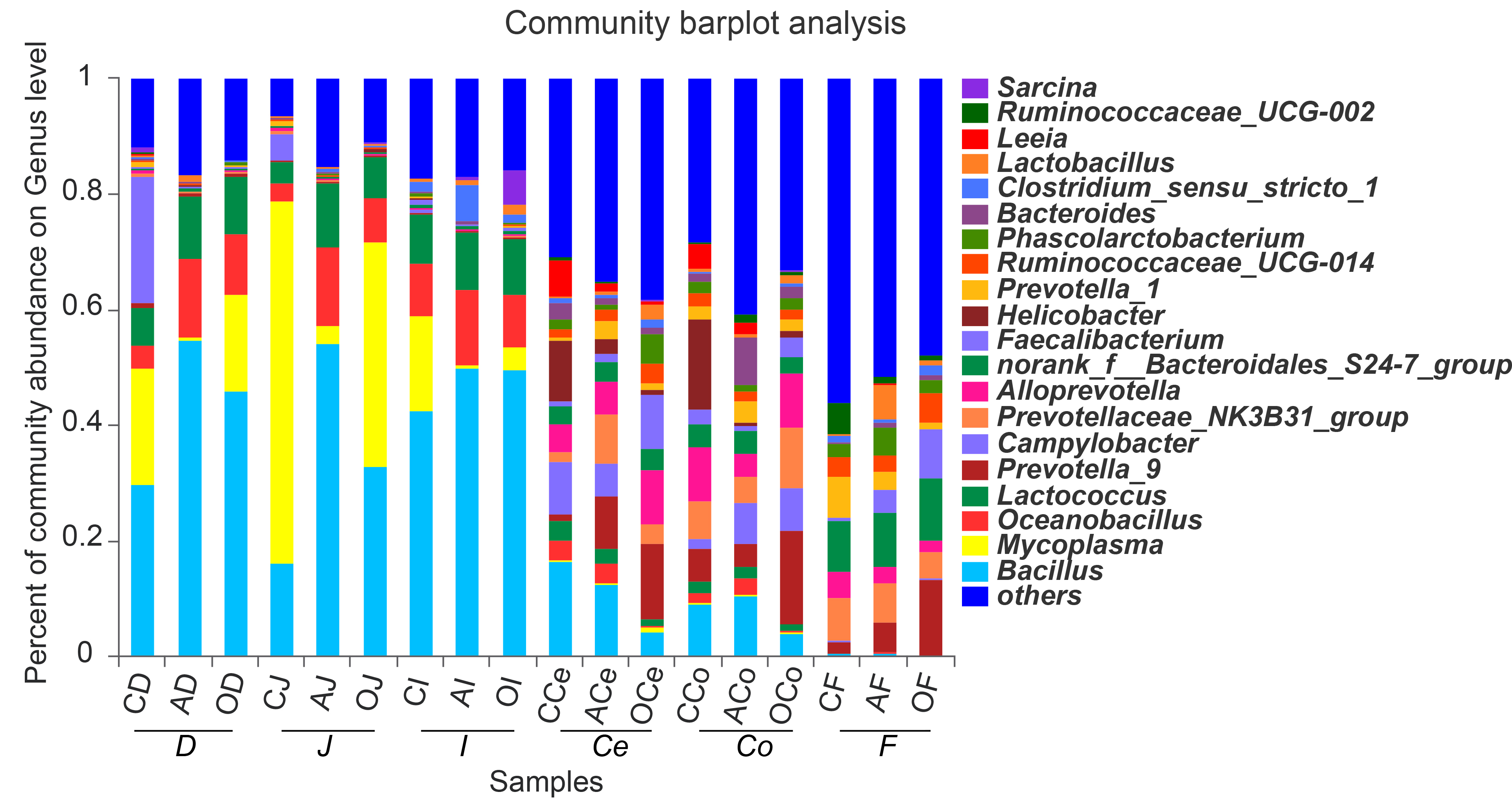

Supplement: Supplementary file 1 [file Data_Sheet_1.ZIP › 20180827_SHI_Supplementary_Material/supplementary_Figure/Figure S4.jpg]

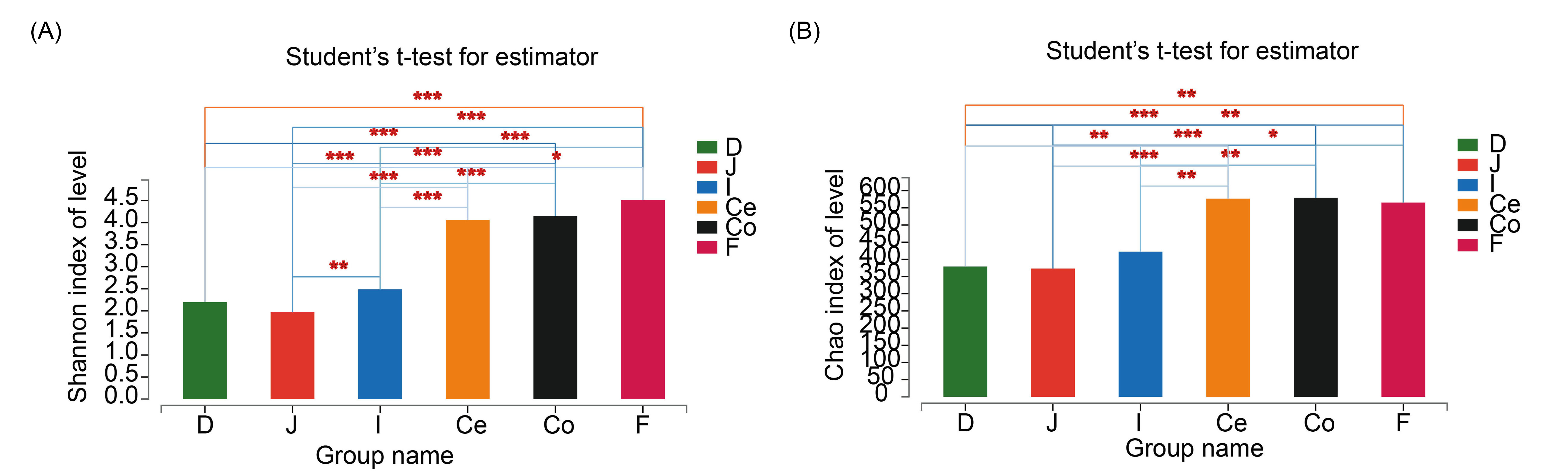

Supplement: Supplementary file 1 [file Data_Sheet_1.ZIP › 20180827_SHI_Supplementary_Material/supplementary_Figure/Figure S5.jpg]

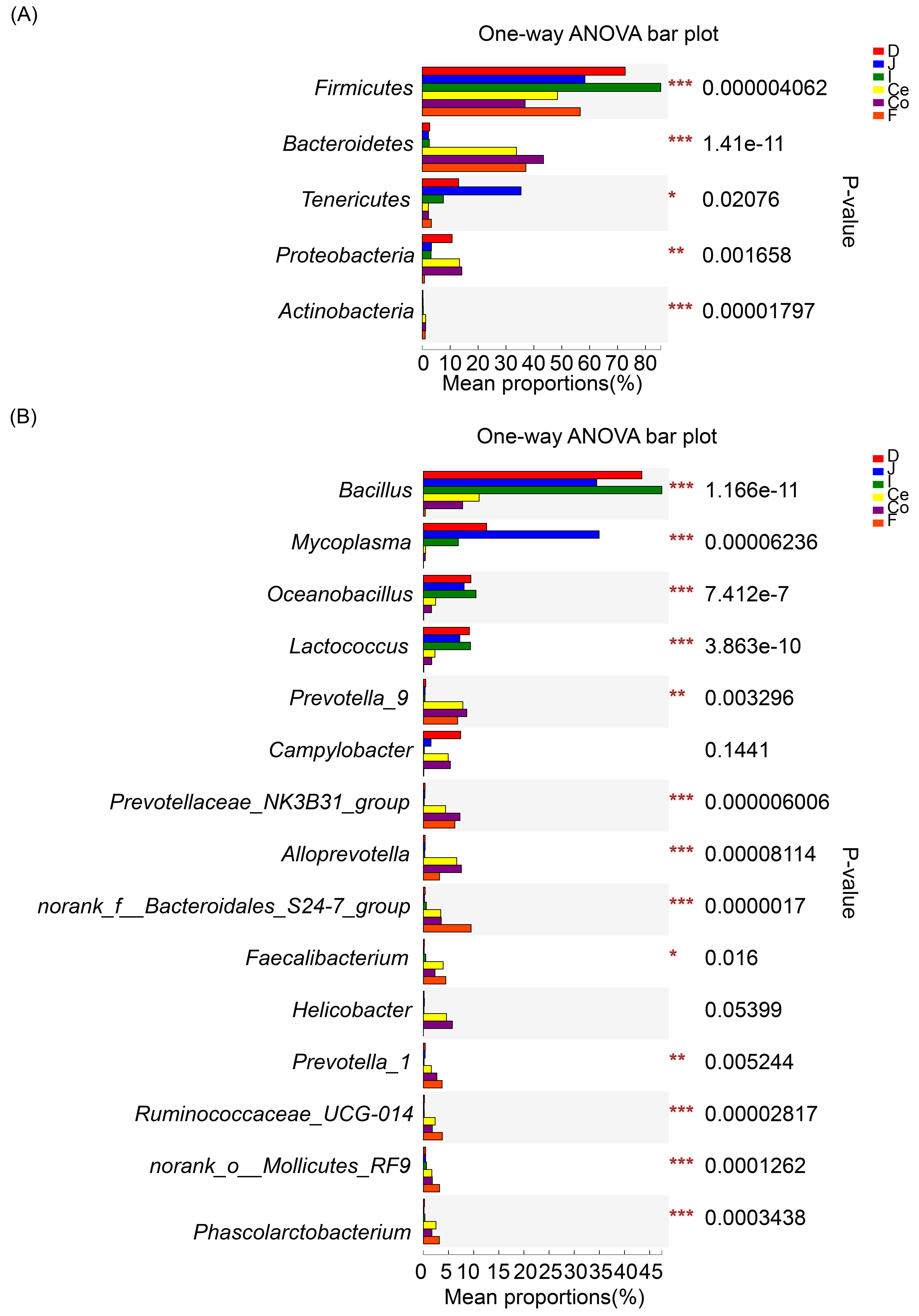

Supplement: Supplementary file 1 [file Data_Sheet_1.ZIP › 20180827_SHI_Supplementary_Material/supplementary_Figure/Figure S6.jpg]

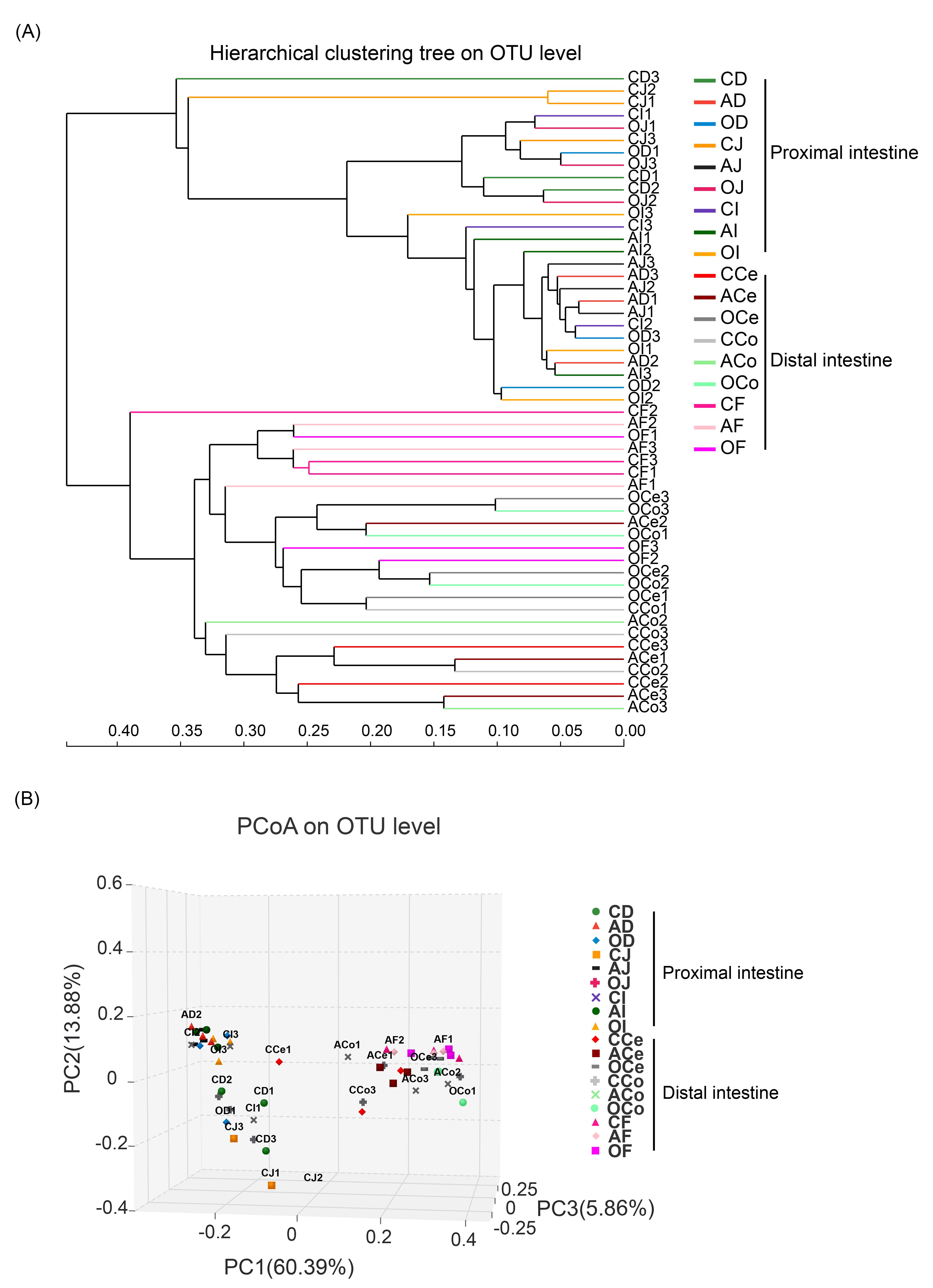

Supplement: Supplementary file 1 [file Data_Sheet_1.ZIP › 20180827_SHI_Supplementary_Material/supplementary_Figure/Figure S7 .jpg]
